# The predictive value of delta-like3 and serum NSE in evaluating chemotherapy response and prognosis in patients with advanced small cell lung carcinoma: An observational study

**DOI:** 10.1097/MD.0000000000038487

**Published:** 2024-06-07

**Authors:** Chenghua Zhu, Jianling Huang, Xiao Jin, Changwen Zhang, Changjun Zhu, Minjie Lv, Sixi Chen, Xingran Du, Ganzhu Feng

**Affiliations:** a Department of Respiratory Medicine, The Second Affiliated Hospital of Nanjing Medical University, Nanjing, Jiangsu, China; b Department of Respiratory Medicine, The Affiliated Jiangning Hospital of Nanjing Medical University, Nanjing, Jiangsu, China.

**Keywords:** chemotherapy response, delta-like 3, neuron-specific enolase, prognosis, small cell lung cancer

## Abstract

Lung cancer is one of the most malignant tumors with fastest morbidity and mortality. Small cell lung cancer (SCLC) is the most malignant pathological type of lung cancer with early metastasis and poor prognosis. At present, there is a lack of effective indicators to predict prognosis of SCLC patients. Delta-like 3 protein (DLL3) is selectively expressed on the surface of SCLC and is involved in proliferation and invasion. Neuron-specific enolase (NSE) is an enolase isoenzyme that is generally regarded as a biomarker for SCLC and may correlate with stage of SCLC, prognosis and chemotherapy response. NSE can be influenced by different types of factors. To explore the associations between expression levels of DLL3 in tumor tissues with platinum/etoposide chemotherapy response, and assess the prognostic values of DLL3, NSE and other potential prognostic factors in advanced SCLC patients were herein studied. Ninety-seven patients diagnosed with SCLC in Zhongda Hospital from 2014 to 2020 were enrolled in the study. Serum NSE levels were tested using ELISA methods before any treatment. The expression of DLL3 in tumor tissue was detected by Immunohistochemistry (IHC). We investigated the relationship of DLL3 expression with chemotherapy and survival. Progression free survival (PFS) and overall survival (OS) were estimated by the Kaplan–Meier method. Multivariate Cox-proportional hazard regression was used to identify predictors of PFS and OS. DLL3 was detected in 84.5% (82/97) of all patients’ tumor samples by IHC, mainly located on the surface of SCLC cells. Lower DLL3 expression was associated with longer PFS and better chemotherapy response. OS had no significant differences. Multivariate analysis by Cox Hazard model showed that, high DLL3 expression and maximum tumor size >5 cm were independent risk factors for PFS, where NSE < 35 ng/mL and age < 70 were independent prognostic factors for OS. Early stage was independent prognostic factors for PFS and OS (*P < *.05 log-rank). DLL3 was expressed in the most of SCLCs. DLL3 expression level in the tumor and NSE level in the serum may be useful biomarkers to predict the prognosis of SCLC. DLL3 may be a potential therapeutic target for SCLC in the future.

## 1. Introduction

Small cell lung cancer (SCLC) approximately accounts for about 15% of all lung cancers and is the most malignant pathological type characterized by low differentiation, rapid proliferation, high invasiveness, and neuroendocrine function.^[1,2]^ Majority of patients have distant metastases at the time of initial diagnosis they lose the chance of surgery. The standard 1st-line treatment for extended-stage SCLC (ES-SCLC) patients is platinum-based chemotherapy, such as cisplatin or carboplatin.^[3]^

Despite being highly responsive to initial chemotherapy and radiotherapy, most patients relapse within 6 months due to development of drug resistance, leading to disease progression.^[4]^ To date, topotecan is the only recommended 2nd-line drug by the FDA for treatment of ES-SCLC and is mainly effective in patients who are also sensitive to 1st-line treatment.^[5]^ Clinical trial results showed that, anlotinib prolonged the median PFS (4.1 vs 0.7 months) and median OS (7.3 vs 4.9 months) in patients who accepted at least 2 lines of previous chemotherapy.^[6,7]^ Based on these positive results, anlotinib has been approved as the 3rd or later line treatment for refractory SCLC in China since August 2019. Impower 133 trial confirmed that, combined atilizumab (immune checkpoint inhibitors, ICIs) to the 1st-line standard chemotherapy prolonged the OS by 2 months in patients with ED-SCLC, and reduced the risk for death by 30%.^[7,8]^ However, most patients, especially those with refractory SCLC, still demonstrate either primary resistance or rapid acquired resistance to ICIs.^[9]^ Despite increased treatment options, the prognosis of SCLC patients is still poor and 5-year survival rate is lower than 7%.^[3]^ Improving the sensitivity of 1st-line chemotherapy and postponing the occurrence of drug resistance may contribute to ameliorated prognosis of SCLC. Therefore, it is important to find effective biomarkers to predict the sensitivity of platinum drugs.

Notch signaling plays a vital regulated role in cell proliferation, differentiation, and apoptosis.^[10]^ DLL3 is an inhibitory ligand of Notch signaling that is specifically expressed on the surface of SCLC cells rather than normal lung tissues, which makes it a potential biological target for SCLC.^[11,12]^ Preclinical studies have shown that, DLL3 promotes the tumorigenesis of SCLC by inhibiting the Notch receptor pathway and activating PI3K/Akt signaling.^[13]^ At present, there is controversy about the relationship between DLL3 levels and prognosis of SCLC. A study has shown that, low DLL3 expression contributed to a longer OS.^[12]^ However, a study including 63 SCLC patients found that, the DLL3 expression level was not correlated to OS.^[14]^ Moreover, other recent research showed that, there was no significant difference between DLL3 expression with disease free survival (DFS) and OS in SCLC patients undergoing surgical treatment. Therefore, whether DLL3 can be a molecular marker for predicting the prognosis of SCLC needs to be confirmed. However, there are few relevant literature reports to explain correlation between DLL3 expression and chemotherapy response.

Neuron-specific enolase (NSE) is an enolase isoenzyme that is extensively located in nerve and neurogenic tumor cells, which plays a key role in aerobic glycolysis. Researchers found the expression of NSE in SCLC cell lines early in the 1980s.^[15,16]^ Serum NSE levels fluctuated in different stages of SCLC, frequently elevated at the initial time of diagnosis, declined after remission, and rebounded after progresses or relapses.^[17–19]^ It made NSE a essential tumor marker for diagnosis and prognosis evaluation of malignancies, including SCLC for a long time.^[20,21]^ However, the prognostic value of NSE in SCLC patients remains controversial according to results from many researches. Nowadays, the NSE has become a widely used and easily attainable laboratory assay in SCLC patients. However, serum NSE level may differ due to its detecting methods, such as enzyme-linked immunosorbent assay (ELISA), electron chemiluminescence immunoassay (ECSIA) and radioimmunoassay assays (RIA).^[22]^ NSE elevation was not only present in malignant tumors but also in some benign diseases, such as brain injury,^[23]^ which may disturb the predictive value of NSE as a biomarker. Therefore, limitations are inevitable that NSE alone as an indicator for predicting the prognosis of SCLC. As reported, a series of molecular biomarkers may be more appropriate to precisely predict the patient’s prognosis than single molecule.^[24,25]^ Hence, this retrospective study was set out to examine the expression of DLL3 in Chinese people diagnosed with advanced SCLC and its relationship with clinical parameters and platinum-based chemotherapy response. We also attempted to integrate DLL3 expression and pretreatment serum NSE for predicting the survival of advanced SCLC. To date, this is the first investigation about utilizing the combination of DLL3 and NSE to predict the prognosis.

## 2. Materials and methods

### 2.1. Study population

Patients first diagnosed with SCLC in Zhongda Hospital from 2014 to 2020 were included in this study. Inclusion criteria were as following: ≥18 years of age; measurable tumors using computerized tomography (CT) scanning; Eastern Cooperative Oncology Group (ECOG) performance status between 0 and 1; predicted survival time ≥ 2 months; had enough samples for ICH. Patients were excluded if they met any of the following criteria: severe cardiovascular and cerebrovascular diseases, liver and kidney dysfunction, and blood system diseases; combined malignancy or recurrent or metastatic tumors; initial treatment was surgery; data including gender, age, smoking history, VALG stages, maximum tumor size, and ki67 index were recorded. Serum NSE levels were tested using ELISA kit (Human NSE ELISA Kit, ZY0370EH, ZeYe, China) before any treatment. All participants received at least 2 cycles of regular platinum-based chemotherapy and regular assessment. The research was reviewed and approved by Ethical Committee of Zhongda Hospital (No. 2018ZDSYLL089-P01).

### 2.2. Expression level

Tumor specimens obtained by fiberoptic bronchoscopy, computerized tomography-guided needle, and Lymph node biopsy at the time of initial diagnosis were chosen to examine the expression of DLL3 by IHC analysis. No cytological material was included. Paraffin-embedded tissues were sectioned at 3 µm and incubated with primary antibody (Rabbit anti-DLL3 monoclonal antibody (E3J5R) #71804, Abcam, China, dilution, 1:100) was conducted at 4 °C for overnight. semiquantitative analysis of the IHC staining was performed independently by 3 trained pathologists who were blinded to all subject clinical characteristics and survival status. We distributed patients into DLL3- high group (scores ≥ 6) and DLL3-low group (scores < 6) according to products (IHC scores = PC * SI) of stained cells percentage (PC, 1 = 1–9%, 2 = 10–49%, 3 = 50–79%, 4 = ≥80%) and maximum staining intensity (SI, 0 = no staining, 1 = weak, 2 = medium, 3 = strong).

### 2.3. Therapeutic response and survival

Tumor response was assessed every 2 cycles of platinum/etoposide chemotherapy according to Response Evaluation Criteria in Solid Tumors 1.1 (RECIST 1.1) criteria, including complete response (CR), partial response (PR), stable disease (SD), and progressive disease (PD). The formulas for calculating response rate (%) (RR) and disease control rate (%) (DCR) were adopted as described before, RR = (CR + PR)/all treated patients × 100% and DCR = (CR + PR + SD)/all treated patients × 100%.

### 2.4. Follow-up

The patients were followed up by outpatient consultation, hospitalization record, and recant telephone. The last follow-up time was December 31, 2021, and main end points were PFS and OS.

### 2.5. Statistical analyses

All analyses were performed with the use of SPSS version 24. Pearson test was used to explore the connection between DLL3 expression, clinical characteristics and chemotherapy response. Kaplan–Meier method was applied for survival analysis and comparisons between groups were analyzed by log-rank test. The Cox-proportional hazard model was utilized for multivariate analysis, and hazard ratios (HRs) obtained were reported as relative risks with corresponding 95% confidence intervals (CIs). All statistical tests were 2-sided and *P* values <.05 were considered statistically significant.

## 3. Results

### 3.1. Baseline characteristics

**Figure 1.**
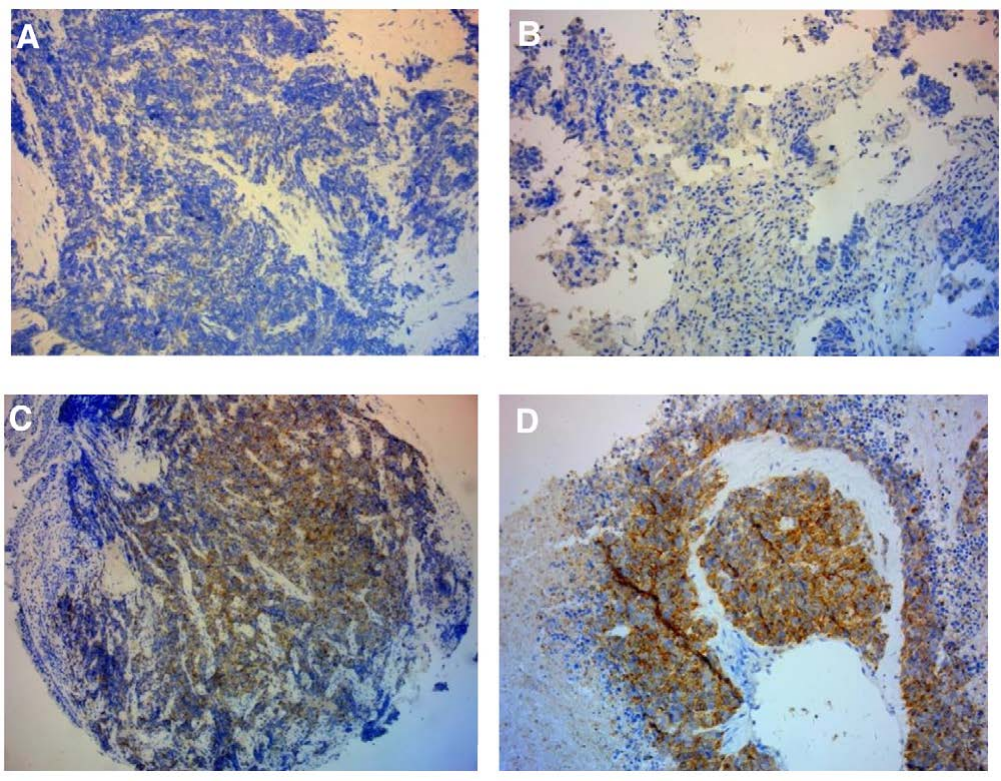
(A–D) Immunohistochemical staining of DLL3 in SCLC specimens (10×). (A) Negative DLL3 expression (IHC Score = 0); (B) low DLL3 expression (IHC Score = 4); (C, D) high DLL3 expression (IHC Score = 8 and 12). DLL3 = delta-like 3 protein, IHC = immunohistochemistry, SCLC = small cell lung cancer.

Basic clinical characters are shown in Table 1. The median age of patients was 66 years (range, 44–89) and majority of patients were male. According to Veterans Administration Lung Study Group (VALG) staging criteria, 35 (36.1%) patients were at limited stage (LD) and 62 (63.9%) patients at extensive stage (ED). The ECOG performance status of patients was almost normal. Current or ever-smokers consisted of 81.4% (79/97) patients. Serum NSE levels in approximately half of the patients were beyond 35 ng/mL. Meta-analysis showed that, the cutoff levels of NSE reported in the studies ranged from 7.5 to 35 ng/mL.^[21]^ We chose 35 ng/mL as the cutoff level in the study. DLL3 was detected in 84.5% (82/97) of all patients’ samples and mainly located on the surface of SCLC cells. 35.1% (34/97) were high expression and 15.5% (15/97) were negative for dyeing (representative images can be seen in Fig. 1). Sixty-three patients were divided into DLL3-low group and the rest of patients were into 2 DLL3-high groups. IHC scores are shown in Table 2. Pearson test showed that, elevated DLL3 was associated with increased serum NSE values but not associated with age, gender, stage, smoking history, tumor size, and ki67 index (also shown in Table 1).

**Table 1:** Clinical characteristics and correlation with DLL3 expression levels.

Characteristic		DLL3 expression		χ^2^	*P*
		High	Low		
Age (yr)					
<70	62	18	44	0.098	.123
≥70	35	16	19		
Gender					
Male	83	31	52	0.726	.366
Female	14	3	11		
Smoking					
Yes	79	30	49	0.981	.277
No	18	4	14		
Stage					
LD	35	11	24	0.164	.824
ED	62	22	40		
Serum NSE					
<35 ng/mL	47	10	37	7.600	**.010**
≥35 ng/mL	50	24	26		
Size (maximum)					
≤5 cm	68	25	43	0.293	.648
>5 cm	29	9	20		
KI67 expression					
≤60%	18	8	10		
>60%	79	26	53	0.857	.416

**Table 2:** The expression of DLL3 in tumor tissues.

IHC Score	0	1	2	3	4	6	9	12
Patients (n)	15	11	15	7	15	22	6	6

### 3.2. Chemotherapy response

Seventy-two patients treated with regular platinum/etoposide chemotherapy were selected to evaluate the chemotherapy efficacy. Chemotherapy responses in DLL3-high (n = 34) versus DLL3-low (n = 63) were as follows: CR in 0% versus 3.2% patients, PR in 35.3% (12/34) versus 58.7% (37/63) patients, SD in 20.6% (7/34) versus 12.7% (8/63) patients, and PD in 44.1% (15/34) versus 25.4% (16/63) patients. The RR in the DLL3-high group was 35.3%, which was lower than DLL3-low group (61.9%, *P < *.05). The DCR rates between the 2 groups were 55.9% versus 74.6%, which had no statistical significance (*P > *.05) (as shown in Table 3). These findings suggested that, patients with lower DLL3 expression have a better response to platinum/etoposide chemotherapy (*P < *.05).

**Table 3:** Chemotherapy efficacy evaluation.

Assessment	DLL3-high (n = 34)	DLL3-low (n = 63)	χ^2^	*P*
CR (n)	0	2	–	–
PR (n)	12	37	–	–
SD (n)	7	8	–	–
PD (n)	15	16	–	–
RR (%)	35.3	61.9	6.272	**.019**
DCR (%)	55.9	74.6	3.559	.071

CR = complete response, DCT = disease control rates, PD = progressive disease, PR *=* partial response, RR = response rate, SD *=* stable disease. RR = (CR + PR)/all treated patients × 100% and DCR = (CR + PR + SD)/all treated patients × 100%.

### 3.3. Prognosis

**Figure 2.**
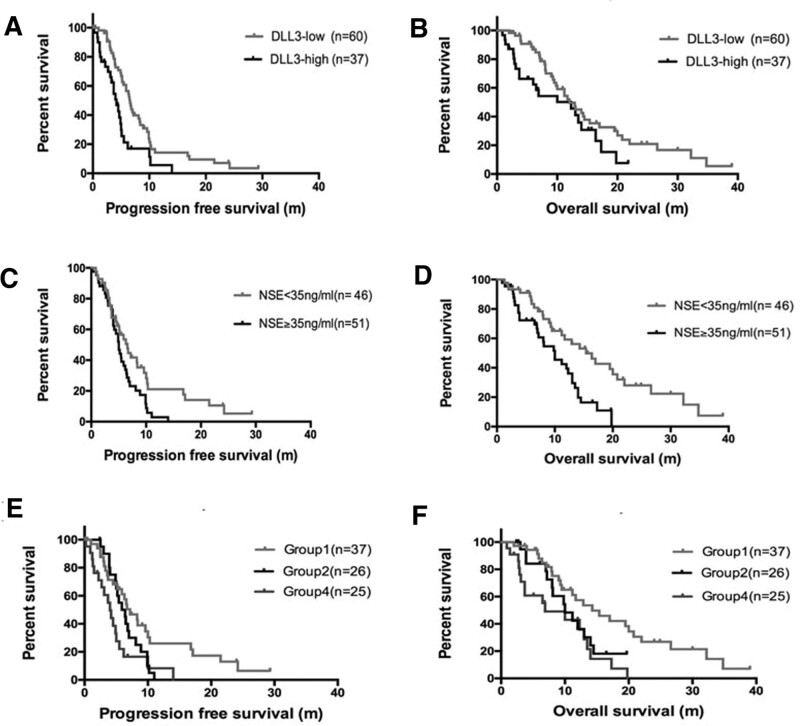
Kaplan–Meier survival curves for PFS and OS. (A, B) Comparison of PFS and OS in total patients according to DLL3 expression levels. (C, D) Comparison of PFS and OS in total patients according to serum NSE levels. (E, F) Comparison of PFS and OS in total patients according to the combination of DLL3 expression and serum NSE levels. DLL3 = delta-like 3 protein, NSE = neuron-specific enolase, OS = overall survival, PFS = progression free survival.

We next compared the survivals of patients in both groups. PFS and OS curves are shown in Figure 2. The median PFS in DLL3-low group was 6.6 months (range: 5.6–7.6) compared with 4.2 months in DLL3-high group (range: 3.1–5.3), which had statistical difference (*P = *.009, log-rank, Fig. 2A). Median OS between the 2 groups was 12 months versus 12.3 months, which had no obvious difference (*P > *.05, log-rank, Fig. 2B). On the other hand, the median PFS in NSE < 35 ng/mL group were 6.6 months (95% CI: 6.062–8.934), and had no statistical difference from the NSE ≥ 35 ng/mL group (5 months, *P > *.05, Fig. 2C). The median OS in the NSE < 35 group was 14.5 months (95% CI: 13.862–21.473), which was significantly longer than that in the NSE ≧ 35 ng/mL group (9.8 months, *P = *.016, Fig. 2D). Moreover, to estimate the combined effect of DLL3 and NSE in influencing the survival of patients, we divided the enrolled patients into 4 groups (Group 1 = DLL3-low + NSE < 35 ng/mL, Group 2 = DLL3-low + NSE ≥ 35 ng/mL, Group 3 = DLL3-high + NSE < 35 ng/mL, Group 4 = DLL3-high + NSE ≥ 35 ng/mL). Because of limitation in sample collection, which was seriously censored (n = 9), Group 3 was not included for survival analysis. The median PFS was, respectively, 7.2, 5.4, and 4.5 months. PFS in group1 was significantly longer than that in Groups 2, 4 (Fig. 2E, *P = *.008). Median OS was 14.3, 8.1, and 10 months, respectively, which had statistic difference between Groups 1, 2, and 4 (Fig. 2F, *P = *.011). These data implied that patients with low DLL3 expression and serum NSE < 35 ng/L presented the best survival rate compared to patients with elevated DLL3 or NSE.

The Cox-proportional hazard model was applied to analyze multivariate factors’ impact on the prognosis of SCLC (shown in Table 4). These results revealed that, high DLL3 expression (HR = 2.122; 95% CI: 1.164–3.868; *P = *.014), female (HR = 0.355; 95% CI: 0.127–0.991; *P = *.048), and maximum tumor size > 5 cm (HR = 0.512; 95% CI: 0.300–0.875; *P = *.014) were independent risk factors for PFS in patients with advanced SCLC. Correspondingly, NSE < 35 ng/mL (HR = 2.676; 95% CI: 1.377–5.200; *P = *.004), lower ki67 expression in tumor tissues (HR = 2.199; 95% CI: 1.038–4.661; *P = *.040), and age < 70 years (HR = 2.206; 95% CI: 1.127–4.318; *P = *.021) were independent prognostic factors for OS. Early stage and regular chemotherapy treatment were independent prognostic factors for PFS and OS (*P < *.001).

**Table 4:** Prognostic factors for PFS and OS by multivariate Cox-proportional hazards regression model.

Variables	PFS		*P*	OS		*P*
	HR	95% CI		HR	95% CI	
Tissue DLL3	2.122	1.164 to 3.868	**.014**	1.123	0.562 to 2.247	.742
Serum NSE	1.182	0.685 to 2.038	.548	2.676	1.377 to 5.200	**.004**
Age	1.338	0.740 to 2.419	.335	2.206	1.127 to 4.318	**.021**
Stage	3.121	1.670 to 5.835	**.000**	2.856	1.448 to 5.633	**.002**
Gender	0.355	0.127 to 0.991	**.048**	0.443	0.166 to 1.185	.105
Tumor size	0.512	0.300 to 0.875	**.014**	1.016	0.577 to 1.787	.957
Smoking	1.866	0.831 to 4.193	.131	1.117	0.467 to 2.667	.804
Tissue Ki67 (%)	1.198	0.626 to 2.291	.585	2.199	1.038 to 4.661	**.040**

## 4. Discussion

This study revealed that, DLL3 and NSE were respectively, significant and independent predictive factors for PFS and OS of SCLC. Lower DLL3 expression in tumor tissue of SCLC was observed with better chemotherapy response and longer PFS. Meanwhile, Lower levels of serum NSE displayed longer OS. In addition, the predictive value of the combination of DLL3 and NSE was better than that of DLL3 and NSE alone.

In our study, we examined the expression of DLL3 in 97 Chinese SCLC patients by IHC and found that, the DLL3 was expressed beyond 80% of patients and was not associated with clinical indices, such as age, sex, smoking history, and clinical stage, which consisted with previous reports.^[14]^ Survival analysis showed that, there was a significant difference in PFS but no significant difference in OS among patients with high and low DLL3 expression. Therefore, the DLL3 expression alone could not be a good biomarker for predicting the prognosis of SCLC. We also found that, the overall RR in the DLL3-high group was lower than in the DLL3-low group, which implied that, patients with DLL3-low expression were more sensitive to chemotherapy. Recent evidence suggested that, the DLL3 may be closely related to proliferation and invasion of SCLC. The most obvious findings in this research were as follows: DLL3 protein was specifically expressed on the surface of most neuroendocrine tumor cells, but was rarely detectable in normal lung tissues. It promoted tumor growth and inhibited apoptosis by activating PI3K/Akt signaling pathway^[14,26]^; DLL3 inhibited Notch activation and might be a direct downstream target for ASCL1, which was related to phenotype of neuroendocrine tumors, and it may play an important role in the occurrence and development of neuroendocrine tumors, especially in SCLC^[15–17]^; Konstantakou showed that, the DLL3 regulated the conversion between mesenchymal to epithelial transition (MET), and epithelial transition to mesenchymal (EMT), confirming its association with SCLC invasion.^[18]^ Besides, Notch signaling and EMT have been verified associated with tumor therapeutic resistance. The DLL3 was considered as a potential therapeutic target for SCLC. At present, several drugs was included in clinical trials, such as RovaT, AMG 757, and AMG 119.^[19,20]^ DLL3 may play a role in therapeutic resistance. Further fundamental experiments are needed to verify this hypothesis.

NSE is an important tumor biomarker for SCLC, and its serum level was closely correlated with tumor progression, recurrence, and chemotherapy effect of SCLC patients.^[27–29]^ However, serum NSE values may differ due to its unequal detecting methods and antibodies. Besides tumors, some other factors could have an influence on NSE concentration, which limits its function as a prognostic indicator. In our study, we found that, DLL3 expression was higher than that in the DLL3-Low group, when compared with low expression in the serum NSE value of patients in the DLL3-high expression group. NSE < 35 ng/mL was an independent prognostic factor for OS but not for PFS. Therefore, we combined DLL3 and NSE to predict the prognosis of patients with SCLC, so that they make up for their personal shortcomings. Results showed that, the low DLL3 expression and serum NSE < 35ng/L had better prognosis than any increased DLL3 and NSE. Thus, combining DLL3 and NSE may be considered as one of the prognostic predictors among patients with SCLC in clinical practice.

This study had several limitations. First, it was a single-center and retrospective research with a relatively small sample size. Second, immune-histochemical staining was semi quantitative analysis whose quantitative results are not accurate enough. In addition, it was greatly affected by experimental operation problems and the doctors’ experience in judging IHC scores, which make the results subjective. Third, no validation cohort was included to verify the findings. Lastly, we had not further investigated the correlation between DLL3 expression and therapeutic resistance. Multicenter and prospective studies with considerably large data are thus needed to testify our findings in the future.

In conclusion, DLL3 expression level was negatively correlated with chemotherapy response. The combination of DLL3 level and pretreatment serum NSE values may be extremely valuable for predicting the prognosis of SCLC. Maximum tumor size < 5 cm, NSE < 35 ng/mL, age < 70 years, early stage could significantly improve the survival of patients with SCLC. These findings might help clinicians to judge disease condition and formulate personalized and reasonable adjuvant therapies for SCLC patients.

## Author contributions

**Writing – original draft:** Chenghua Zhu, Jianling Huang, Xingran Du, Ganzhu Feng.

**Writing – review & editing:** Chenghua Zhu, Jianling Huang, Ganzhu Feng.

**Validation:** Jianling Huang, Sixi Chen, Xingran Du, Ganzhu Feng.

**Formal analysis:** Xiao Jin.

**Investigation:** Changwen Zhang.

**Methodology:** Changwen Zhang.

**Project administration:** Changjun Zhu.

**Resources:** Changjun Zhu.

**Software:** Minjie Lv.

**Supervision:** Minjie Lv, Ganzhu Feng.

**Visualization:** Sixi Chen, Xingran Du, Ganzhu Feng.
